# Relation Between Circulating Vitamin K1 and Osteoporosis in the Lumbar Spine in Syrian Post-Menopausal Women

**DOI:** 10.2174/1874312901812010001

**Published:** 2018-01-22

**Authors:** Sawsan Jaghsi, Taghrid Hammoud, Shaden Haddad

**Affiliations:** 1 Department of Biochemistry and Microbiology, Faculty of Pharmacy, Damascus University, Damascus, Syria; 2 Department of Physiology and drugs, Faculty of Medicine, Damascus University, Damascus, Syria

**Keywords:** Vitamin K, Bone Mineral Density, Osteoporosis, Postmenopausal Women, Lumber Spine, ELISA

## Abstract

**Background:**

In the past two decades, Vitamin K has been receiving more attention due to its role in bone health and metabolism. The bone mineral density does not remain steady with age, particularly declining after menopause.

**Objective:**

This study is aimed to investigate the relationship between bone mineral density and serum vitamin K1 levels in post-menopausal women, and to evaluate serum vitamin K1 levels as a potential biomarker for postmenopausal osteoporosis.

**Methods:**

Serum levels of vitamin k1 were measured in 23 postmenopausal osteoporotic women, and in 15 postmenopausal healthy control women using a standardized Enzyme-Linked Immune Sorbent Assay (ELISA) kit. Bone mineral density BMD was assessed at the lumbar spine.

**Results:**

The mean serum vitamin k1 level was significantly lower in the postmenopausal osteoporotic women group than in the normal control group (mean=0.794 vs3.61ng/ml, P< 0.0001), and serum vitamin k1 concentration was positively correlated with lumbar spine BMD among postmenopausal osteoporotic women (R=0.533, p = 0.009), and in postmenopausal healthy control (R=0.563, p = 0.02).

Diagnostic sensitivity and specificity of vitamin k1 for osteoporosis were 90% and 98%, respectively (cut-off value: 0.853 ng/ml). The area under the ROC curve (AUC) value for vitamin k1 was 0.984 the odd ratio result was 18.66.

**Conclusion:**

Our results suggest that vitamin K1 may contribute to maintain bone mineral density. Vitamin K1 may have a role in diagnosing post-menopausal osteoporosis. Vitamin K1 may be a valuable diagnostic as well as therapeutic marker in post-menopausal osteoporosis.

## 1 INTRODUCTION

Osteoporosis is a metabolic bone disease of reduced bone density, fragile bone and heightened vulnerability to fracture [1]. Risk factors for osteoporosis include genetic, hormone and nutrition [2]. Bones are living tissue and constantly changing. Bone remodeling is a succession of bone resorption by osteoclasts and bone formation by osteoblasts. In an adult, osteoclastic bone resorption and osteoblastic bone formation usually happen at the same rate and it keeps the bone mass constant.

Bone mineral density loss with age and osteoporosis is due to exceeding osteoclastic bone resorption without the parallel quantity of bone formation by osteoblasts.

In women, bones reach their maximum density between 20 and 30 years of age, peak bone mass remains at the same level until the age of around 40 years, and then it gradually decreases up to the menopause, after which it drops [3].

Vitamin K is getting more attention in relation to its role in bone metabolism besides its role in blood clotting. Vitamin K plays a biological role as a cofactor of gamma carboxylase, which mediates γ- carboxylation of glutamic acid residues (Glu) to γ-carboxyglutamic acid (Gla) on vitamin K-dependent protein . The Ƴ-carboxylation of the vitamin K-dependent proteins Gla proteins is essential for their function.

Some of the vitamin K-dependent proteins exist in bone such as osteocalcin (bone Gla protein) and Matrix Gla Protein (MGP). Osteocalcin needs vitamin K for its mineral-binding capacity related to adding mineral to the bone matrix in normal bone growth and development [2].

Vitamin K is shown to improve bone mineralization and decrease bone resorption by osteoclasts [4]. Other Vitamin K roles have also been reported such as it can promote fracture reparation by stimulating bone formation, and decrease calcium excretion by urine [2]. These results confirm the significant role of vitamin K in bone metabolism.

Vitamin K is actually a group of compounds which include vitamin K1(phylloquinone) and vitamin K2 (menaquinone). Vitamin K1 is obtained from leafy greens and some other vegetables. Vitamin K2 is a group of compounds which are called menaquinone-n (MK-n) largely obtained from meats, cheeses, eggs, and synthesized by bacteria [5].

Dietary recommendations (120µg/day for men, 90µg/day for women) are based on saturation of the coagulation system. Requirements to maintain bone Gla proteins function and bone formation might be higher [6].

In the present study, we compared serum levels of vitamin K1 between osteoporotic women and healthy control women, and we also investigated the relationship between levels of vitamin K1 with bone mineral density in postmenopausal women.

## 2 MATERIALS AND METHODS

### 2.1 Patients and Samples

Our study is a prospective cross-sectional study. This study included two groups. Group 1, had 23 postmenopausal osteoporotic women (with reduced bone mineral density BMD) (OSP), group 2 had 15 healthy control postmenopausal non-osteoporotic women (with normal BMD at all of lumber spine, femoral neck and hip) (nOSP). The groups were considered as age – matched groups (age did not differ significantly between the groups). Women in the two groups were within (3-5 years) postmenopausal, Patients with secondary causes of bone loss such as renal failure, malignancy, gastrointestinal abnormalities, thyroid diseases, parathyroid diseases, arthritis, or osteomalacia were excluded, and subjects under treatment with drugs known to affect bone metabolism (estrogen, glucocorticoids, anti- convulsants, vitamin D, calcium), as well as subjects under treatment with vitamin K antagonists, vitamin k supplement were also excluded. All of these samples were collected between Januray 2015 and April 2016 at Damascus Hospital.

The study was approved by Ethical commission of Damascus University, and written informed consent was obtained from all patients when they were enrolled.

### 2.2 Methods

Sample collection: Fasting blood were obtained from all women in clinical standard serum tubes. Whole blood was centrifuged at 3000 rpm (1509×g) for 20 min, and aliquots were stored in light- protected conditions at -80ºC.

#### 2.2.1 Measurement of Serum Vitamin K1 Level

Vitamin K1 levels were measured by sandwich ELISA method using a commercial kit (Sun Red, China) according to the manufacturer’s instructions and spectro photo microplate reader at Damascus University (Elysisuno - Human, Germany).

#### 2.2.3 Measurement of Bone Mineral Density

Osteodensitometry: bone mineral density (BMD) was Measured in lumber spine (L2-4) by using Dual Energy X-ray Absorptiometry (DEXA)

### 2.3 Statistical Analysis

The distribution of vitamin K1was first tested within the groups, and then the difference was evaluated in vitamin K1 concentrations between the two different groups compared by Mann-Whitney U test.

The dependent variable was BMD measured at lumbar (L2-**-**L4) spine. We assessed the linearity of the relationships between measures of BMD and the independent variable serum vitamin K1 (in patients

and control as one group) using linear regression methods. The data fit nonparametric curves. The resulting curves suggested a linear relationship between BMD and vitamin K1. Thus, we fit linear regression models to the data. Correlation between serum levels of vitamin K1 and BMD in each group was analyzed using Spearman’s correlation coefficient

ROC Curve analysis was used to evaluate the diagnostic value. The optimal cutoff values were calculated using the maximum sum of sensitivity and specificity, and area under curve was calculated (0.5 being no discrimination, 1.0 being ideal discrimination). In addition to sensitivity and specificity, the odds ratio for potential of classification of (OSP) versus (nOSP) was calculated indicating the possibility of a correct diagnosis if this test is applied. Statistical analyses were conducted using IBM SPSS Statistics 20 and Microsoft Excel 2010.

The results were considered statistically significant when the P value was < 0.05.

## 3 RESULTS

### 3.1 Subject Characteristics

Subject characteristics are summarized in Table **1**.

### 3.2 Serum levels of vitamin K1

**Fig. (1):**
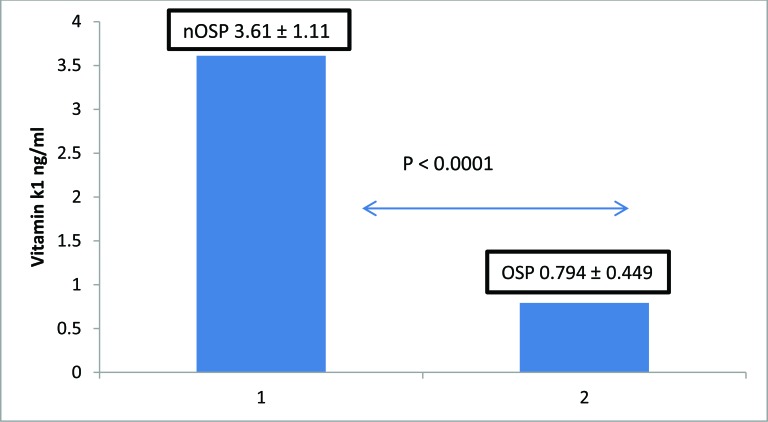
Serum levels of vitamin K1 in post-menopausal women distributed according to their bone mineral density. (OSP): osteoporotic women group (n = 23). (nOSP): normal bone mineral density group (n =15). The mean serum vitamin K1 level in (OSP) was significantly lower than in (nOSP) (p-value <0.0001 by Mann-Whitney U test).

The mean concentration (± SD) of serum vitamin K1 in the normal bone density group was 3.61 ± 1.11 ng/ml in the osteoporotic group was 0.794 ± 0.449 ng/ml. Post-menopausal women with osteoporosis had significantly lower levels of vitamin K1 than women with normal bone density (P<0.0001), No significant differences were observed with respect to their biological parameters ALP, Ca, P, 25-hydroxy vitamin D. (Fig. **1**)

### 3.3 Association between Serum Vitamin K1 and BMD

#### 3.3.1 Correlation between Serum Vitamin K1 and BMD of Spine Among Post-Menopausal Women with Osteoporosis

**Fig. (2):**
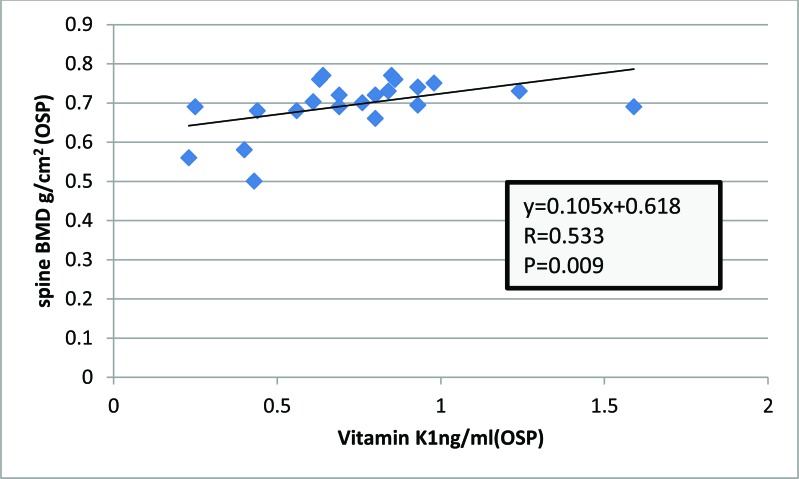
Association between serum vitamin K1 and BMD of the spine among postmenopausal osteoporotic women (R=0.533, p-value = 0.009) by Spearman’s correlation coefficient.

Among (OSP) there were positive association between serum vitamin K1 and BMD of the spine (R= 0.533, P=0.009) (Fig. **2**)

#### 3.3.2 Correlation between Serum Vitamin K1 and BMD of Spine Healthy Control Postmenopausal Women

**Fig. (3):**
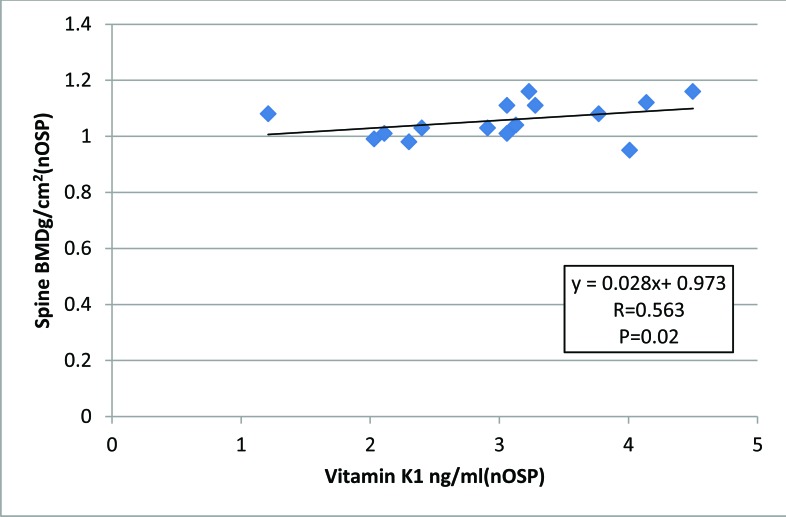
Association between serum vitamin K1 and BMD of the spine among healthy control postmenopausal non-osteoporotic women (R=0.563, p-value=0.02) by Spearman’s correlation coefficient.

Among (nOSP) there were also positive association between serum vitamin k1 and BMD of the spine (R= 0.563, P=0.02) (Fig. **3**).

#### 3.3.3 Multiple Regression Model between Serum Vitamin K1 and BMD Among all Participant Women

Multiple linear regression analysis was performed to evaluate the associations between measures of BMD and serum vitamin K1; the models included terms for serum 25(OH)D, Ca, age. After adjustment for multiple covariates, serum vitamin K1 was positively associated with BMD at the spine in postmenopausal women.

Among all participant women (OSP and nOSP together as one group) there were positive association between serum vitamin K1 and BMD of the spine (R=0.883, R^2^=0.799, P<0.0001), with each increase in 1 ng/ml of serum vitamin K1, there was an increase of 0.115 g/cm2 in BMD at the lumber spine.

### 3.4 ROC analysis

ROC Curve was performed and corresponding AUC was calculated. Vitamin K1 reached AUC (0.984) with sensitivity and specificity of serum vitamin K1 levels in (OSP) group relative to the (nOSP) group were 90 % and 98%, respectively, at a cutoff value of 0.853ng/ml.

Fig. (**4**) shows the ROC curve for serum vitamin K1. The area under the curve (AUC) for serum vitamin K1 was (0.984) 95% P<0.0001.

### 3.5 odd ratio:

**Fig. (4):**
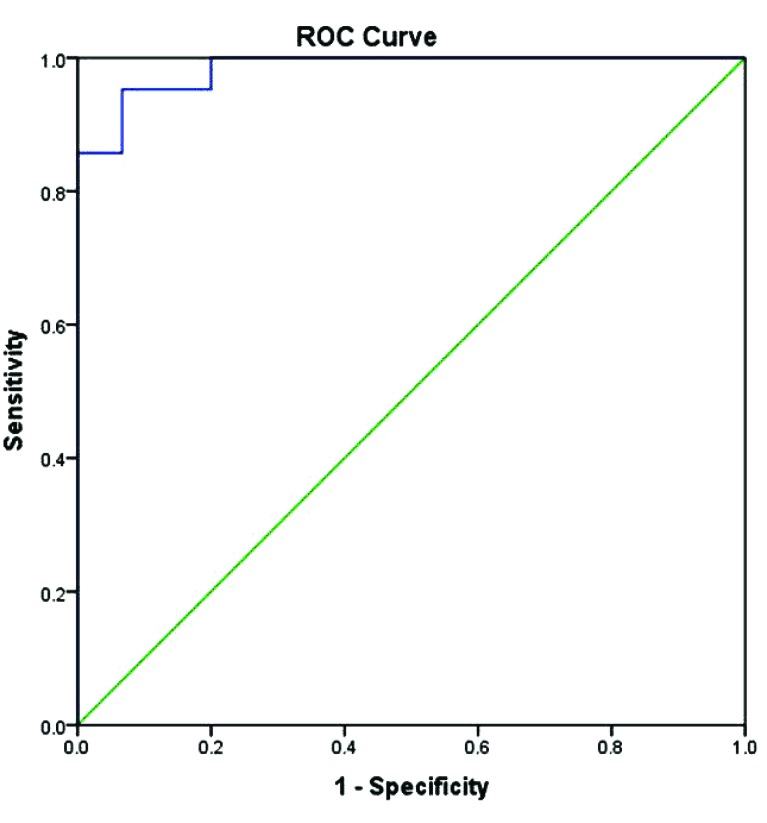
ROC analysis for serum vitamin K1. The area under the curve (AUC) for serum vitamin K1 was (0.984) 95% P<0.0001.

The odd ratio for vitamin was 18.66 with Confidence interval 95% (Fig. **4**).

## 4 DISCUSSION

The skeleton is a metabolically active organ that undergoes continuous remodeling throughout life. Menopause plays a major role in bone loss and subsequent fracture risk in adult women. The basic cause of bone mass loss among postmenopausal women is the reduction in the synthesis of estrogen, which is a natural bone resorption inhibitor in the reproductive age. Therefore, the inadequate compensatory increases in bone formation to offset the increase in bone resorption is an important cause of bone loss in early postmenopausal women [7]. This imbalance is basically attributable to normal processes of aging of bones as well as to genetic and constitutional factors, reduced estrogen levels following menopause, abnormal metabolism of Ca-regulating hormones, unhealthy diet, and lifestyle factors (*e.g*. smoking and insufficient physical activity). At menopause, bone resorption increases more than bone formation, as a result, bone resorption, with an increased number of osteoclasts, is intensified in bone tissue, and bone turnover is activated, leading to increased osteocalcin secretion from osteoblasts and active bone resorption and formation [3]. Osteocalcin has three glutamic acid (Gla) residues which undergo gamma carboxylation by a process dependent on vitamin K, the Ƴ-carboxylation of osteocalcin is necessary for its function. Ƴ-carboxylated osteocalcin shows a high affinity to hydroxyapatite and bone matrix, contributing to bone formation [2]. Studies also reported that vitamin K prevents bone resorption through a mechanism totally different from that of Ƴ-carboxylation [8].

In our study, we found that serum vitamin K1 level was significantly lower in the postmenopausal osteoporotic women group than in the normal control group. The same result was reported by (T. Kanai **et al*.* 1997 their subjects were 19 osteoporotic postmenopausal, mean age 53.9 ± 0.8 years women and 52 non-osteoporotic postmenopausal women mean, age 54.8±1.3 [9]), and (Heiss **et al*.* 2004 their subjects were 8 osteoporotic postmenopausal, mean age 68± 4 years women and 11 non osteoporotic postmenopausal, mean age 66±4 years women [10]). These findings suggested an important role for vitamin K in bone metabolism. And therefore it has been thought that it might be effective for treating osteoporosis.

A cross-sectional study (3199 middle-aged Scottish females included) demonstrated that females in the highest quartile of dietary intake of phylloquinone (VK1) (162 mcg/day) have a significantly higher lumbar spine (L2–L4) BMD and left femoral neck BMD against the lowest quartile (59 mcg/day) [11]. Hara **et al** showed that in in vitro experiments, vitamin K inhibits bone resorption induced by IL-la, PGE, PTH, and vitamin D3 in a dose-dependent manner [12].

Furthermore, serum vitamin K1 concentration was positively correlated with spine BMD in postmenopausal women. This data was in agreement with (Booth **et al**. 2004 [13]), who found that low plasma phylloquinone concentrations were associated with low spine BMD among postmenopausal women not using estrogen replacement. These findings suggest a protective role of vitamin K in the skeleton in women.

High incidence of vertebral fractures has reported to be contrarily correlated with BMD of lumber spine and vitamin k1 concentration in the study on 379 Japanese women of 30–88 years to 4 years [14]. This is consistent with our results.

A study by (Fujita **et al** 2012) [15] reported that high intake of natto, fermented soybean high in phylloquinone, and MK-7 was associated with higher BMD, which was also reported by (Ikeda, Y **et al** 2006) [16].

In an *in vivo* osteoporosis model, Vitamin K was found to increase bone mineral density. A study by (Hodges **et al**) found that osteoporotic patients had decreased levels of vitamin K and increased levels of non Ƴ- carboxylated osteocalcin [17].

ROC analysis of serum vitamin k1 Levels in (OSP) in comparison with (nOSP):

According to ROC analysis, vitamin K was an important parameter to classify nOSP and osteoporotic women (OSP) with a sensitivity rate of 90% and specificity of 98%, at a cutoff value of 0.853 ng/ml. (AUC) for vitamin k1 was 0.984, 95% P <0.0001

In addition, the odd ratio for vitamin was 18.66 with Confidence interval 95%. This data was in agreement with (Heiss **et al** 2004 [10]) who found that vitamin K was the best single parameter to classify nOSP and osteoporotic women with a sensitivity rate of 64% and specificity rate of 82%, and the odds ratio for vitamin K was 16.7.

The results confirm the importance of vitamin K for bone metabolism in postmenopausal osteoporosis. Then, studies showed that the administration of vitamin K led to an increase of bone mineral density in osteoporotic patients, (Vermeer **et al**. 1995 [18]). In addition, administration of vitamin K was found to prevent bone loss and reduce the incidence of fractures (Shiraki **et al**. 2000 [19])

A survey investigated vitamin K intake data from the fifth Korea National Health and Nutrition Examination Survey, which was included subjects of (2785 men, 4307 women) aged over 19 years, reported that low dietary vitamin K intake was related to low bone mineral density in subjects. In addition, there was a reduction in risk for osteoporosis as vitamin K intake increased in women, but this effect was not continued after adjusting factors. This survey recommended increasing the dietary VK intakes for preserving BMD [20].

The relations between vitamin K intake and Bone Mineral Density (BMD) are not consistent in observational studies [21].

Fang *et al* deduced that vitamin K supplements did not have influence on BMD at femoral neck, but there was an increase in mean lumbar spine BMD by 1.3% (95% CI: 0.5–2.1) after 6–36 months of supplementation.

In this meta-analysis, seven studies utilized vitamin K1 with portions ranging from 0.2 to 10 mg/day. Ten studies utilized vitamin K2 (eight used MK-4 with portions of 15–45 mg/day, and two studies utilized MK-7 with portions of (0.2–3.6 mg/day) and after studies with high risk of bias have been excluded, the writer deduced that supplementation with vitamin K did not have significant effect on lumbar spine BMD in their subgroup analysis, they found that supplementation with vitamin K2 increased mean lumber spine BMD by 1.8% (95% CI: 0.9–2.8). No such influence was realized for studies with vitamin K1 supplementation [22].

It has been reported that osteoporosis is linked with oxidative stress. Moreover, supplementation of VK, as an antioxidant vitamin, could effectively reduce levels of oxidative stress, with possibly advantageous influence on bone, as displayed in several experimental models [8].

Vitamin K is necessary for bone health. In fact, low VK intake, low VK circulating levels, and high under carboxylated osteocalcin levels are all related with excessive hip fractures risk in observational studies [23].

Studies approved a relation between lessened intake of VK1 and low BMD in women, but on the whole, there is smaller proof for the relation between intake of VK and increase of BMD. Disagreements in methodological quality of the chosen trials, differences in the type of vitamin K utilized and differences in reports of sufficiency of calcium and vitamin D intake, wide differences in populations of study, and publication bias were restrictions of these studies [8]. Some of the interventional studies on effects of vitamin K1 on bone BMD in postmenopausal women are summarized in Table **2**.

However, a limitation of our study is the small sample size. At the time the samples were collected, it was rather difficult to obtain sample from women with osteoporosis without suffering from the above mentioned exclusion diseases such as thyroid diseases, arthritis, or osteomalacia, and that they are not under treatment with drugs known to affect bone metabolism. More studies are required regarding the influences of vitamin K on diagnosis and management of osteoporosis, and likewise on BMD.

## CONCLUSION

Our study suggests that vitamin k may contribute in maintaining BMD and diagnosing osteoporosis in postmenopausal women.

## Figures and Tables

**Table 1:** Parameters and factors that affect bone metabolism in post-menopausal women distributed according to their BMD.

Characteristic	Healthy Control Group (nOSP) (n=15)	Osteoporotic group(OSP) (n=23)
Age	55.9 ± 0.3	56.8 ± 1.3
BMD (g/cm^2^)L2-4	1.05 ± 0.066	0.693 ± 0.072
BMI (kg/m^2^)	35.53 ± 4.29	33.21± 5.31
Vitamin D (ng/ml)	30.08 ± 3.52	28.8 ± 5.53
Calcium (mg/dl)	9.1 ± 0.34	9.28 ± 0.47
Phosphor (mg/dl)	3.25 ± 0.47	3.12 ± 0.4
ALP (IU/L)	77.33 ± 36	82.06 ± 41

Table (**1**). subject characteristics (mean ± SD)

**Table 2:** Summary of the results of some randomized controlled trials examining the effects of vitamin K1 supplementation on BMD in postmenopausal women:

**Reference**	**Type of study**	**Country**	**Participants**	**Type and dose of** **vitamin K**	**Duration**	**Results**
Bolton-Smith C *et al*. 2007 [24]	Randomized controlled studies	UK	Healthy postmenopausal Women (n=209)	K1 (200mcg/day) and/or vitamin D (400 IU/day) plus calcium (1000 mg/day) vs. placebo.	Two years	ultradistal radius BMD and BMC was increased in K1 plus vitamin D plus calcium group vs. placebo
Kanellakis S *et al*. 2012 [25]	Randomized controlled studies	Greece	Postmenopausal women (n=219)	K1 or K2 (100 mcg/day) vs. placebo	one year	Total BMD was increased in all groups vs. placebo, increased BMD of lumbar spine vs. placebo after adjusting for changes in serum vitamin D concentration and dietary calcium intake
Moschonis G *et al*. 2011 [26]	Randomized controlled studies	Greece	Postmenopausal women (Ca vit.D group, n = 26) (Ca vit.D vit.K1 group, n = 26) (Control group, n = 39)	K1 (100mcg/day) or K2 (100 mcg/day) vs. placebo. Fortified milk and yoghurt, one control group on normal diet.	One year	Total BMD was increased vs. placebo
